# Fluorescent-Nanoparticle-Impregnated Nanocomposite Polymeric Gels for Biosensing and Drug Delivery Applications

**DOI:** 10.3390/gels9080669

**Published:** 2023-08-18

**Authors:** Kumaraswamy Gandla, K. Praveen Kumar, P. Rajasulochana, Manoj Shrawan Charde, Ritesh Rana, Laliteshwar Pratap Singh, M. Akiful Haque, Vasudha Bakshi, Falak A. Siddiqui, Sharuk L. Khan, S. Ganguly

**Affiliations:** 1 Department of Pharmaceutical Analysis, Chaitanya (Deemed to be University), Hyderabad 500075, India; 2 Department of Pharmaceutical Chemistry, School of Pharmaceutical Sciences, Government of NCT of Delhi, Delhi Pharmaceutical Sciences and Research University (DPSRU), New Delhi 110017, India; 3 Department of Microbiology, Saveetha Medical College and Hospitals, Saveetha Institute of Medical and Technical Sciences, Saveetha University, Kanchipuram 602105, India; 4 Department of Pharmaceutical Chemistry, Government College of Pharmacy, Karad 415124, India; 5 Department of Pharmaceutics, Himachal Institute of Pharmaceutical Education and Research (HIPER), Hamirpur 177033, India; 6 Department of Pharmaceutical Chemistry, Narayan Institute of Pharmacy, Gopal Narayan Singh University, Rohtas 821305, India; 7 Department of Pharmaceutical Analysis, School of Pharmacy, Anurag University, Hyderabad 500088, India; 8 Department of Pharmaceutics, School of Pharmacy, Anurag University, Hyderabad 500088, India; 9 Department of Pharmaceutical Chemistry, N.B.S. Institute of Pharmacy, Ausa 413520, India; 10 Department of Pharmaceutical Chemistry, School of Pharmacy, Anurag University, Hyderabad 500088, India; 11 Bar-Ilan Institute for Nanotechnology and Advanced Materials, Ramat Gan 5290002, Israel

**Keywords:** fluorescent nanoparticles, gels, biomedical applications, biosensing, imaging

## Abstract

Nanocomposite polymeric gels infused with fluorescent nanoparticles have surfaced as a propitious category of substances for biomedical purposes owing to their exceptional characteristics. The aforementioned materials possess a blend of desirable characteristics, including biocompatibility, biodegradability, drug encapsulation, controlled release capabilities, and optical properties that are conducive to imaging and tracking. This paper presents a comprehensive analysis of the synthesis and characterization of fluorescent-nanoparticle-impregnated nanocomposite polymeric gels, as well as their biomedical applications, such as drug delivery, imaging, and tissue engineering. In this discourse, we deliberate upon the merits and obstacles linked to these substances, encompassing biocompatibility, drug encapsulation, optical characteristics, and scalability. The present study aims to provide an overall evaluation of the potential of fluorescent-nanoparticle-impregnated nanocomposite polymeric gels for biomedical applications. Additionally, emerging trends and future directions for research in this area are highlighted.

## 1. Introduction

Nanocomposite polymeric gels are intricate substances that amalgamate the characteristics of the polymer matrix and nanoparticles, resulting in a distinctive material with improved properties [1]. The polymer matrix functions as a structural support for the nanoparticles, which are methodically dispersed within the substance [2]. The characteristics of nanocomposite polymeric gels are influenced by various factors, such as the nature and amount of nanoparticles, the properties of the polymer matrix, and the processing parameters employed during the fabrication process [3,4,5]. The incorporation of metallic nanoparticles has been observed to augment the electrical conductivity of the material. Similarly, the inclusion of magnetic nanoparticles has been found to facilitate the material’s ability to react to an external magnetic field [6,7,8,9]. The capacity of nanocomposite polymeric gels to manifest various properties simultaneously, including mechanical robustness and biocompatibility, represents a significant advantage. The aforementioned properties render them highly suitable for employment in biomedical domains, including but not limited to tissue engineering, drug administration, and wound management.

Nanocomposite polymeric gels have been employed in the advancement of sensors and actuators owing to their capacity to react to exogenous triggers such as temperature, pH, or light [10,11]. A hydrogel that has been impregnated with gold nanoparticles exhibits the ability to undergo chromatic alterations as a reaction to variations in temperature, thereby rendering it a valuable component in the realm of temperature sensing [12]. Nanocomposite polymeric gels possess distinctive characteristics that render them highly appealing for employment in environmental remediation [13]. Specifically, they can be tailored to selectively adsorb or degrade pollutants, thereby enhancing their utility in this domain [14]. A hydrogel that incorporates iron oxide nanoparticles has the potential to effectively eliminate heavy metals from polluted water [15,16,17,18].

Current research endeavors are concentrated on devising novel techniques for the synthesis and characterization of these substances, in addition to investigating their potential utility in nascent domains such as energy storage and conversion [19,20,21,22].

The distinguishing optical and physicochemical properties of fluorescent nanoparticles have garnered considerable interest in contemporary times as a form of nanomaterial [23]. The aforementioned characteristics are a result of the nanoscale nature of the materials, which contain a fluorescent dye or quantum dot that is restricted to a limited volume. This confinement results in significant, size-dependent interactions with light [24]. The optical characteristics of fluorescent nanoparticles, such as their luminosity, durability, and emission range, can be adjusted by modifying the nanoparticle’s composition, the fluorescent dye or quantum dot employed, and the particle’s dimensions and configuration [25]. This facilitates the production of fluorescent nanoparticles possessing distinct optical characteristics that are well suited for particular applications [26]. In biological imaging, fluorescent nanoparticles have been used to label and track cells, tissues, and biomolecules, allowing researchers to observe and study biological processes at the cellular and molecular levels. Fluorescent nanoparticles have been utilized in biosensing applications to detect and quantify specific analytes, such as proteins or nucleic acids, in complex biological samples [27]. Fluorescent nanoparticles have been utilized in the realm of environmental monitoring to identify and measure pollutants and toxins present in the environment. Furthermore, fluorescent nanoparticles have been employed in various fields, such as optoelectronics, energy conversion, and as optical probes, for investigating materials at the nanoscale. Although fluorescent nanoparticles have numerous advantages, there exist apprehensions regarding their safety and probable toxicity [28]. Current research endeavors are directed towards comprehending the plausible hazards linked with the utilization of fluorescent nanoparticles and devising tactics to mitigate these risks [29]. These strategies encompass enhancing biocompatibility and diminishing toxicity [30].

The application of fluorescent nanoparticles and nanocomposite polymeric gels exhibits significant potential for a diverse array of biomedical applications [31,32,33]. The foremost benefit of this methodology lies in its capacity to design materials possessing distinctive optical and mechanical characteristics, rendering them highly suitable for diverse biomedical purposes [34]. Fluorescent nanoparticles that are incorporated into nanocomposite polymeric gels have the potential to be utilized for precise drug delivery and imaging purposes. Fluorescent nanoparticles possess the capability of being functionalized with specific ligands or antibodies, thereby enabling them to selectively target cancer cells or other disease targets [35]. The nanocomposite polymeric gel matrix has the capability to be tailored for the purpose of releasing the drug payload in reaction to particular stimuli, including pH, temperature, or light. This feature allows for meticulous management of drug release [36]. The utilization of fluorescent nanoparticles in conjunction with nanocomposite polymeric gels represents a potent technique for visualizing biological tissues and cells. The nanoparticles’ luminosity, which is vivid and strong, can be employed for the purpose of marking and monitoring cells or biomolecules in real time. This enables scientists to observe and analyze biological mechanisms at the cellular and molecular levels [37]. The utilization of fluorescent nanoparticles incorporated within nanocomposite polymeric gels presents the advantage of facilitating in vivo imaging and monitoring of therapeutic responses. Fluorescent nanoparticles possess the capability to be deliberately designed in a manner that allows them to emit light at specific wavelengths. This characteristic facilitates their detection and monitoring within living organisms through the utilization of non-invasive imaging techniques, such as fluorescence imaging [38]. The application of fluorescent nanoparticles incorporated into nanocomposite polymeric gels is generally regarded as a promising approach for a wide range of biomedical applications. These applications include targeted drug delivery, in vivo imaging, and assessment of therapeutic responses [39]. The ability to alter materials so that they possess unique optical and mechanical properties presents a powerful mechanism for the development of innovative therapeutic interventions and diagnostic devices for a wide variety of diseases. The fabrication of microdroplets for a glucose biosensor, known as the fluorescent hydrogel glucose biosensor (FHGB), was achieved through the utilization of a microfluidic technique. This method involved the use of glass capillaries with a coaxial flow-focusing geometry [40]. The resulting microdroplets were composed of cross-linked poly(acrylic acid) (PAAc), which had been immobilized with carbon dots (CDs), glucose oxidase (GOx), and horseradish peroxidase (HRP). This immobilization process occurred following the conversion of poly(acrylamide) to PAAc. Due to their biocompatibility, these droplets have the potential to be utilized as implanted continuous-detection biosensor devices. Using glucose, xylose, and glucosamine as starting materials, Wang et al. synthesized various biomass CQDs and made CQDs/ALg and CQDs/CNF luminous hydrogels [41]. The composite hydrogels exhibited favorable fluorescent characteristics and notably enhanced mechanical properties as a result of the cross-linking influence of carbon quantum dots (CQDs).

The objective of composing a review article on such topic is to present a thorough and inclusive summary of the present-day advancements in this nascent area of investigation. This paper aims to provide an overview of recent developments in the creation and production of luminescent nanoparticles embedded in nanocomposite polymer gels for medical purposes. These applications include the precise delivery of medication, real-time imaging within living organisms, and the observation of the efficacy of therapeutic interventions.

## 2. Classification of Fluorescent Nanoparticles

Nanoscale clusters of organic or inorganic crystalline or amorphous materials that produce light at specified wavelengths are known as fluorescent nanoparticles (FNPs). Over the past twenty years, FNPs have seen substantial research and development as well as medical and technology implementation. Unlike many other fluorescent materials, such as dyes, FNPs are more stable, absorb and emit light more efficiently, and have longer fluorescence lifetimes. Unlike dyes, multiple FNPs with distinct emission wavelengths can be utilized in tandem. FNPs are also multimodal, meaning that the dots can serve many purposes at once. A single dot, for instance, can serve as a fluorescent marker to indicate successful delivery of a therapeutic ingredient to cancer tissue. For particle tracking at the cellular level, FNPs provide a non-invasive alternative to isotopic techniques. FNPs have not been widely accepted in fundamental biological research despite that their features make them suitable for tracking, detecting, and visualizing animals and molecules. In the early 1980s, seminal publications were published describing semiconductor nanoparticles [42], and by the late 1990s, cadmium chalcogenide quantum dots (Cd-containing QDs; Cd-QDs) were successfully used in cell imaging. There have been significant advances in the introduction of new technologies in the nanoscale world since the creation of the first QDs. The shells, coatings, dopings (additions of atomic impurities during synthesis), and conjugations used with Cd-QDs have resulted in significant enhancements to the material’s optical and chemical characteristics, biocompatibility, and practical applications. Additionally, non-cadmium FNPs based on metal, metalloid, and carbon cores have been produced. These are just now beginning to make their way into the biosphere. These new advancements pave the way for the widespread use of this technology in areas such as ecology and physiology. Researching the food web structure, predator prey preferences, plant and fungal seed and spore dispersal dynamics, microbial network nutrient cycling dynamics, and microbial movement and behavior patterns in soil and water may all be carried out with the help of the latest generation of FNPs.

For nanocrystals made of semiconductors, semiconductor quantum dots glow when exposed to light of a specified wavelength. Semiconductors are defined as materials with a medium electrical conductivity. The small size of the dots restricts the motion of the electrons, causing them to reside at distinct levels of energy, hence the name “quantum confinement effect”, on the properties of QDs. Particle size is no longer a confounding factor in elaborate experiments because the emission color may be adjusted by changing the relative abundances of different elements in the core or on the surface [43]. For simplicity, we will refer to the most well-known FNPs as “cadmium quantum dots”, or Cd-QDs for short. Cd-QDs are semiconductor nanocrystals that typically incorporate the chalcogenides, such as sulfur (S), selenium (Se), or tellurium (Te), as companion anions. Typically, the diameter of these QDs is 2–10 nm, but it can be as large as 20 nm [44]. As the shell makes the core cadmium less chemically and biologically available and improves other features, such as photostability and biocompatibility, quantum dots coated with a shell of less-toxic material, such as, for example, zinc sulfide (ZnS), are now the standard type used in biological research [45].

Similar in size to quantum dots (QDs), fluorescent carbon nanoparticles (FCNPs) have a carbon core instead of a semiconductor one and mostly contain the elements oxygen (O), nitrogen (N), and hydrogen (H) [46]. Depending on their synthesized and organized form, FCNPs go by a number of different names. Particles with widely varying characteristics are often referred to by obscure terminology, such as “carbon dot (CD)”. As different research organizations claim to have produced novel varieties of particles, new names and abbreviations are frequently proposed [47]. FCNPs with appropriate surface ligands have promising use as diagnostic tools and medication carriers because of their tiny size and natural composition, which allows them to efficiently target specific tissues. Due to its high molecular mass, the blood–brain barrier (BBB) makes it difficult to administer medications or nanoparticles to the central nervous system, yet CQDs conjugated to transferrin can do so in zebrafish [48].

## 3. Fabrication of Fluorescent-Nanoparticle-Impregnated Nanocomposite Polymeric Gels

### In Situ Gelation Technique

**Figure 1:**
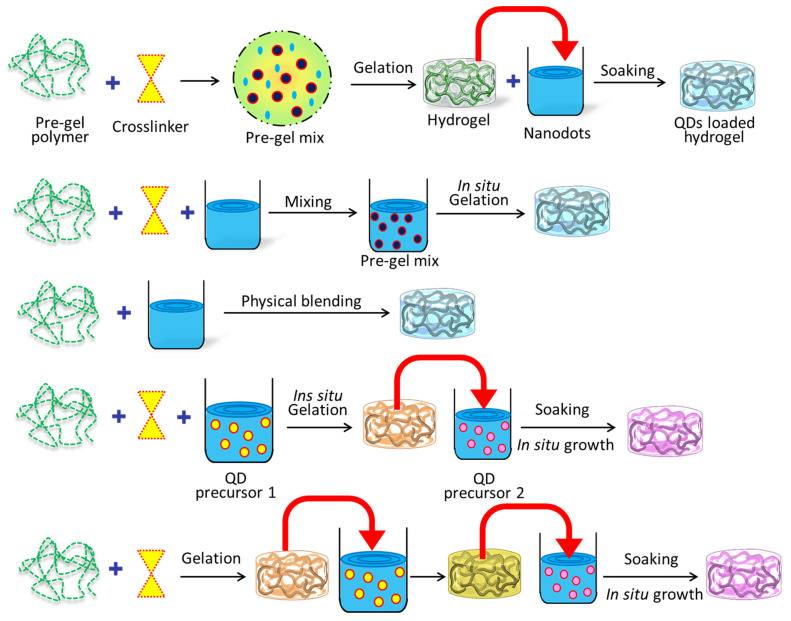
Several distinct methods were utilized in the production of hybrid hydrogels based on QDs.

Several disciplines, including pharmaceuticals, biology, and materials science, have found success with the potential in situ gelation technology [49]. Gels are formed locally, at the point of application, in response to a predetermined set of stimuli. This method is useful for localized medication delivery because it permits regulated and sustained drug release [32,50,51,52,53,54]. Advantages of the in situ gelation procedure include simple administration, fewer systemic adverse effects, and enhanced patient compliance [55,56,57]. It is also a promising platform for targeted therapeutics and biological applications because of its adaptability to a variety of environmental conditions. Researchers have shown considerable interest in the application of quantum dots (QDs) in diverse domains, including biological diagnosis and imaging, optoelectronics, and sensors [58,59,60,61,62,63]. As a result, there has been an increasing interest in developing methodologies for the production of hybrid materials consisting of quantum dots (QDs) and hydrogels, which can exhibit customizable optical properties [32,64]. The synthesis of quantum dots (QDs) involves the initiation and development of crystals, whereas the fabrication of hydrogels encompasses the physical or chemical interconnection of polymeric chains to attain a three-dimensional network architecture [65]. Both procedures correspond to discrete phases in the progression of two separate components. The molecule in question is responsible for an intriguing correlation between two processes that are fundamentally distinct from one another [66]. In the realm of practical applications, numerous challenges have been confronted by researchers. These hurdles encompass the insufficient compatibility between quantum dots (QDs) and hydrogels, as well as the degradation of the optical or electrical properties of QDs upon their conjugation with hydrogels [67,68,69,70]. The dependable incorporation of quantum dots (QDs) into a hydrogel matrix is an essential technological breakthrough. This article provides an analysis of the most recent synthetic approach utilized in the fabrication of quantum dot (QD) and hydrogel hybrids, with particular emphasis on the sequential arrangement of constituent components. This perspective is highly relevant in practical contexts. Figure 1 visually represents the three distinct methodologies. Table 1 shows different types of fluorescent-nanoparticle-loaded hydrogel synthesis methods.

**Figure 2:**
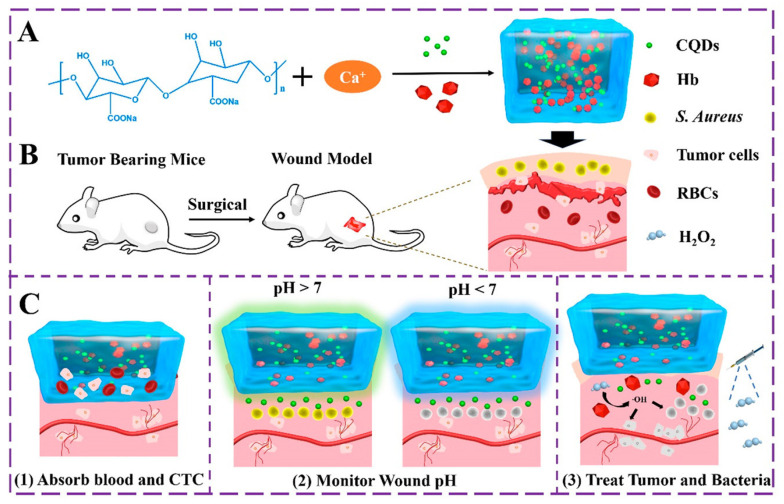
Schematic diagram of: (**A**) synthesis of SA@Hb@CQDs, (**B**) establishment of the postoperative tumor model and the corresponding status of the wound, and (**C**) the innovative CDT-mediated implant nanoplatform for the postoperative treatment of tumors. Reproduced with permission from [81] © 2020 American Chemical Society.

Hydrogels can be prepared using homopolymers or copolymers through chemical or physical cross-linking methods, which impart them with distinct mechanical and chemical properties [40]. Diverse quantum dots (QDs) have been embedded into the bulk hydrogel framework to prepare various QDs-hydrogel composites. Various techniques have been utilized to achieve a homogeneous distribution, including hydrogel gelation in quantum dots (QDs) solution, embedding pre-prepared QDs into hydrogels post-gelation, in situ formation of QDs within pre-formed gels, and cross-linking via QDs to generate hydrogels. The initial step in the fabrication of hydrogels involved the incorporation of the carbon nanodot (C-dot) into polyvinyl alcohol (PVA) through the utilization of the freeze–thaw technique [80]. The C-dot exhibits favorable characteristics, such as optimal dimensions and an abundance of surface functional groups, rendering it a suitable nucleating agent for PVA crystallization. This, in turn, facilitates the formation of a more compact and homogeneous cross-linked network in PVA hydrogel, ultimately resulting in the augmentation its mechanical properties. The incorporation of CDs into PVA hydrogel results in a significant enhancement of the material’s tensile strength, by 46.4%, which is better in comparison to pure PVA hydrogel. These findings suggest that the C-dot is a promising additive for improving the mechanical properties of PVA hydrogel. In addition, the incorporation of CDs into PVA hydrogel has the potential to confer novel characteristics, including fluorescence and reducibility. A rational design has been implemented to create a sodium alginate (SA) hydrogel that is multifunctional in nature, as it immobilizes both hemoglobin (Hb) and pH-sensitive fluorescent changing carbon quantum dots (CQDs) [81]. The focus was on the multi-functionalization of the SA@Hb@CQDs hydrogel, which enables simultaneous detection, hemostasis, and chemo-dynamic therapy (CDT) while monitoring the wound pH based on CQDs (Figure 2). As an innovative CDT-mediated implant nanoplatform, the sodium alginate@hemoglobin@CQDs hydrogel (SA@Hb@CQDs) could be locally implanted in the tumor site for an extended period of time to carry out the detection therapy and then be degraded. It possesses a number of distinct advantages.

**Figure 3:**
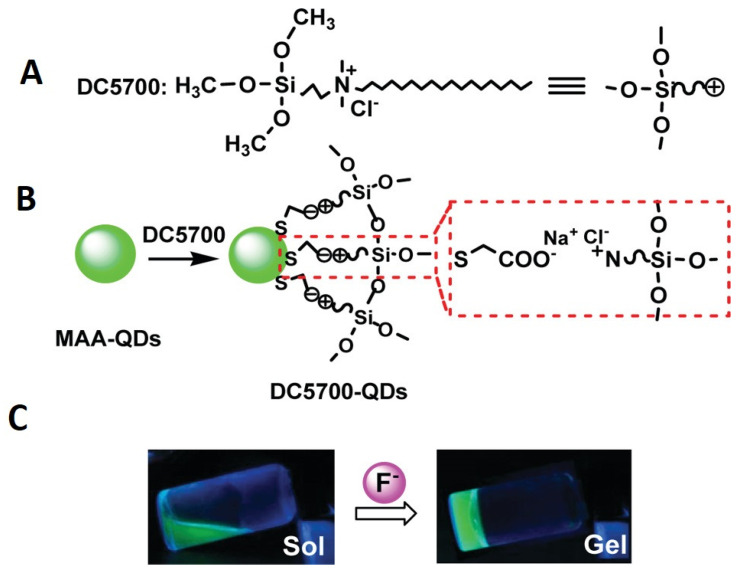
(**A**) Chemical structure of DC5700, (**B**) preparation of DC5700-QDs via ion exchange, and (**C**) sol–gel phase transition of DC5700-QDs solutions treated with F^–^ (fluoride sodium) under an ultraviolet lamp (excitation wavelength = 365 nm). Reproduced with permission from [83] © 2012 American Chemical Society.

By adding a tyrosinase-QDs conjugate solution to the monomer mixture, Park et al. fabricated a tyrosinase-QDs conjugate hydrogel composite [82]. Due to the free-radical polymerization of methacrylate groups of poly(2-hydroxyethyl methacrylate) and poly(ethylene glycol) dimethacrylate, highly cross-linked networks capable of entrapping the tyrosinase-QDs conjugate were formed upon exposure to ultraviolet light. Zhou et al. also used this method to obtain hydrogels embedded with CdTe QDs using a commercial coupling agent named DC5700, followed by a mixture with a variety of anion solutions, and subsequently preparing CdTe QDs [83]. As illustrated in Figure 3, the DC5700-QDs were synthesized through electrostatic interactions involving carboxylate anions and ammonium cations.

The siloxane group exhibits a high degree of sensitivity towards fluoride ion, thereby leading to a swift sol–gel transition upon its induction by fluoride ion, as depicted in Figure 3B. This transition serves as an indication of the hydrogel’s formation. Nonetheless, the amalgamation persisted as a sol upon introduction to additional anionic solutions, thus indicating that this approach could potentially offer a novel means of detecting fluoride ions via sol–gel transition. Notwithstanding its convenience, this approach exhibits a conspicuous limitation whereby the quantum dots (QDs) may exude from the hydrogel matrix in instances of low cross-linking density.

## 4. Infusion of QDs into Hydrogel Technique

The incorporation of quantum dots (QDs) within hydrogels has arisen as a propitious method with a wide range of potential applications. The integration of the distinctive optical features of quantum dots (QDs) with the favorable attributes of hydrogels offers a means to create sophisticated materials that possess customized functionalities. The procedure entails the integration of quantum dots (QDs), commonly in colloidal configuration, into a hydrogel scaffold during its fabrication. Quantum dots (QDs) are semiconductor materials at the nanoscale level that exhibit fluorescence that is dependent on their size and can have their emission wavelengths adjusted. Hydrogels, in the meantime, offer a moist and biocompatible milieu. The hydrogels infused with quantum dots (QDs) exhibit improved optical characteristics and have potential applications in diverse areas such as optoelectronics, biosensing, and biomedical engineering. Furthermore, the utilization of the QD-infused hydrogel methodology facilitates the regulated discharge of enclosed substances, thus rendering it a propitious strategy for drug conveyance systems [84]. Additional investigation and refinement of this methodology may facilitate significant progress in the fields of materials science and biotechnology. The integration of nanoparticles into three-dimensional hydrogel architectures to create nanocomposites is a beneficial approach for augmenting mechanical characteristics or reactivity to specific stimuli. Hydrogel nanocomposites have been utilized in various fields owing to their synergistic effect, with noteworthy emphasis on their biomedical applications [85]. Numerous research groups have integrated quantum dots (QDs) into hydrogels as a means of conserving the nanoparticle and conferring photoluminescence (PL) onto the resultant nanocomposite [86,87].

## 5. Low-Molecular-Weight Polymer-QDs’ Assembly

The combination of low-molecular-weight gelators (LMWGs) and QDs presents a promising avenue for the production of hydrogels, which can yield materials with improved functionalities. The integration of LMWGs, which exhibit self-assembly into fibrous architectures, and QDs, which possess distinctive optical and electronic characteristics, has the potential to yield hydrogels with enhanced mechanical robustness, conductivity, and sensitivity to external stimuli [88]. The hydrogel fabrication process utilizing LMWG-QD assembly encompasses several essential stages. The careful selection of a suitable low-molecular-weight gelator (LMWG) and QDs is of paramount importance [89]. The LMWG should possess the capacity for autonomous assembly into a gel network, thereby conferring the requisite structural integrity to the hydrogel [90,91,92]. In addition, it is essential for the QDs to exhibit favorable characteristics, including the ability to adjust emission wavelengths, a high quantum yield, and the capability for surface functionalization. In the event that an appropriate LMWG is not readily accessible, it may become imperative to engage in the process of synthesizing it. The process of synthesis commonly entails the creation of a precursor molecule that has the ability to undergo self-assembly, resulting in the formation of the intended gelator structure [93,94]. The gelator’s chemical structure and functional groups can be modified in order to achieve compatibility with the QDs and promote their integration into the hydrogel network. The preparation of QDs constitutes a crucial element in the overall fabrication procedure [95]. Commercially available quantum dots can be acquired or synthesized through different techniques, including colloidal synthesis or epitaxial growth. In order to achieve compatibility with the gelator, it is common practice to surface-functionalize the QDs with ligands or polymers. The aforementioned functional groups confer stability, solubility, and promote interactions with the gelator molecules during the process of assembly. The assembly process of the gelator-QD system can be accomplished using diverse methodologies, which are contingent upon the characteristics of both the gelator and the QDs. One methodology entails the combination of the gelator and QDs within a solvent, subsequently proceeding with a meticulously regulated self-assembly procedure. The selection of a suitable solvent is of utmost importance, as it must possess the capability to dissolve both the gelator and QDs without exerting any adverse effects on their assembly or stability.

The process of gelator and QD self-assembly can be influenced by a range of factors, such as hydrogen bonding, electrostatic interactions, π-π stacking, and hydrophobic interactions. The incorporation of QDs into the gelator network has the potential to strengthen the structure, improve mechanical properties, and possibly introduce novel functionalities to the hydrogel. The hydrogel that is formed can display distinct properties as a result of the combined attributes of the gelator and QDs. As an illustration, the incorporation of QDs into hydrogels can confer optical characteristics, such as fluorescence or phosphorescence, rendering them valuable for various sensing or imaging purposes. Furthermore, the inclusion of QDs within the hydrogel matrix has been shown to significantly augment its electrical conductivity, thereby facilitating its utilization in a wide range of electronic devices and biosensors. The synthesis of hydrogels by combining LMWGs and QDs holds great potential for the advancement of functional materials. The integration of the gelator’s self-assembly capabilities and the distinctive attributes of QDs presents novel opportunities for the development of hydrogels with customized properties. The potential applications of this interdisciplinary field extend to various areas, such as biomedicine, tissue engineering, drug delivery, and other related fields.

**Figure 4:**
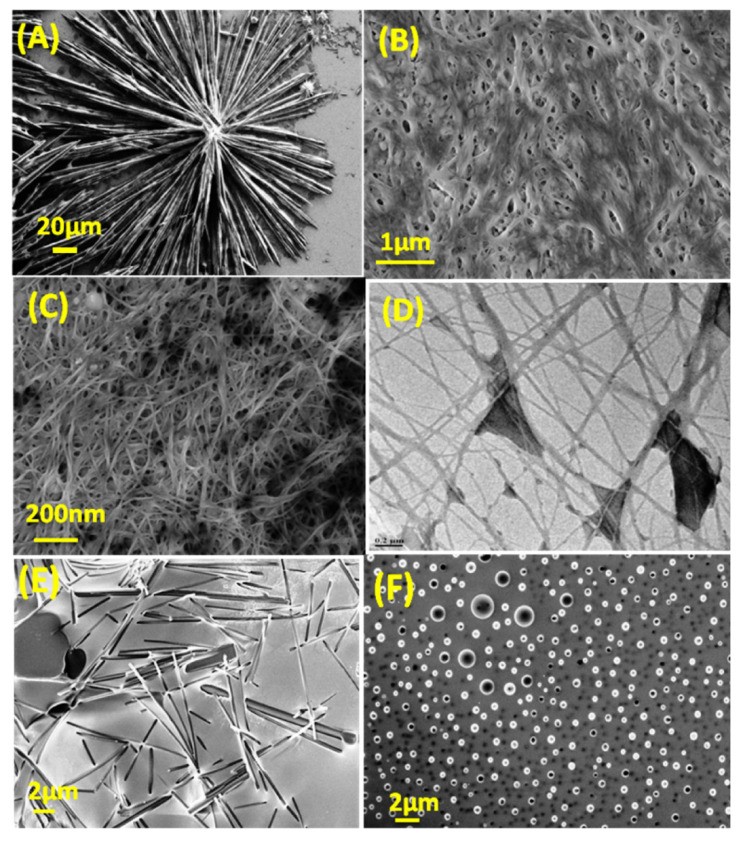
(**A**,**B**) SEM images of peptides P1 and P2 were obtained at the minimum gelation condition of 6 mg/mL. (**C**,**D**) The scanning electron microscopy (SEM) and transmission electron microscopy (TEM) images depict the peptide P3 hydrogels. (**E**,**F**) Electron microscopy (EM) images were obtained for peptides P4 and P5 under the minimum gelation condition, specifically at a concentration of 6 mg/mL. Reproduced with permission from [104] © 2017 American Chemical Society.

The self-assembly of CDs and low-molecular-weight gelators (LMWGs) has attracted significant attention in the scientific community. This method has gained popularity due to its numerous advantages, including its cost-effectiveness, wide applicability, and ability to be reversed [78,79]. The category of LMWGs is extensive, comprising various compounds, such as amides, nucleobases, dendrimers, and fatty acids, among others. LMWGs present the potential for tailoring hydrogel properties according to individual preferences. Quaranta et al. conducted a study wherein they employed scintillators to generate a slender xerogel coating composed of carbon-based nanodots [96]. The incorporation of quantum dots (QDs) into organic or hybrid networks facilitates the extensive utilization of their optical characteristics, ranging from luminescent systems employed in photovoltaics to optical computing applications. Due to their inherent resistance to radiation, colloidal quantum dots (CQDs) possess potential utility in scintillation devices. This concept is quite stimulating. Due to their extensively delocalized structure, colloidal quantum dots (CQDs) exhibit a remarkable ability to endure high-intensity laser irradiation without undergoing degradation [97]. Cayuela et al. synthesized CQDGs using an LMWG, and the authors provide a comprehensive analysis of the effectiveness of these hybrid materials as innovative, durable, rapid, and user-friendly fluorescence sensors [98]. Gels exhibit a significant enhancement in fluorescence as well as exert a substantial influence on selectivity. The surface carboxyl groups present on CQDGs have demonstrated a high degree of selectivity in the detection of Ag^+^ ions. The incorporation of CDs not only enhanced the mechanical strength of the hydrogels, but also resulted in an increased luminosity of the CDs. In the subsequent phase, composite gels were fabricated by employing CDs featuring diverse surface functional groups, namely passivate-CDs with amino groups, thiol-groups, and carboxylic acid groups. Sahub et al. developed a composite hydrogel system utilizing graphene quantum dots (GQDs), enzymes, and small-molecule hydrogelators to enable the detection of organophosphates with high sensitivity and practical utility [99]. During the fabrication process, acetylcholinesterase (AChE) and choline oxidase (ChOx) were incorporated into the self-assembled GQDs/gel materials, resulting in the formation of enzyme-functionalized gel networks. The fluorescence of the graphene quantum dots (GQDs) present in the hydrogels was effectively suppressed by the hydroperoxide generated through the dynamic enzymatic reaction. The fluorescence of GQDs/Enz/gels was observed to be reinstated following exposure to the pesticide dichlorvos. The exhibited sensitivity of these hybrid hydrogels in detecting oxy-form organophosphate pesticides holds promising implications for the prospective advancement of rapid and environmentally sustainable methodologies. The incorporation of functionalized carbon nanodots into a novel hydrogel derived from 5-aminosalicylic acid has been reported [100]. The introduction of heavy-metal ions elicits distinct photoluminescent responses in CDs when exposed to an aqueous environment [101]. The novel hybrid CD-gel nanostructures demonstrated in this study exhibit considerable potential as versatile and customizable luminous materials, offering promising prospects for the detection of heavy-metal ions in environmental and biomedical contexts. Nanostructures fabricated using non-toxic precursors possess the inherent capability of being biocompatible, thereby presenting opportunities for their application in the fields of diagnostics, drug activation, and controlled drug release. By employing colloidal quantum dots (CQDs) as fundamental units, Sun et al. demonstrated the feasibility of fabricating fluorescent vesicles and chiral hydrogels. The formation of vesicles is facilitated by the electrostatic interaction between positively charged CQDs and negatively charged biosurfactant NaDC. This approach eliminates the requirement for time-consuming synthesis, as well as the use of undesirable chemical solvents and complex procedures [102]. The concentration of NaDC was elevated during the fabrication of chiral hydrogels using reduced peptide GSH. The presence of colloidal quantum dots (CQDs) in a gel not only enhances the gel’s brightness but also enhances its mechanical strength. Interestingly, the xerogels also demonstrate strong emission, providing a straightforward method for producing readily deployable phosphors. These phosphors have subsequently been developed into a dependable sensing platform for rapid, on-site detection of Cu^2+^. In a separate study, the researcher employed continuous-flow microfluidics as a means to generate hydrogels and hydrogel/QD-hybrid materials using dipeptides in a continuous manner. The microfluidic system employed in our study facilitated the reliable confinement of inorganic QDs with different sizes and organic porphyrins within a hydrogel formed through supramolecular assembly using Fmoc-FF [103]. The utilization of in situ microfluidic channel monitoring allowed for the observation of a dynamic process in which quantum dots (QDs) were progressively trapped, resulting in the creation of a hybrid hydrogel characterized by consistent QD entrapment. The expansion of potential applications for hybrid gels, specifically dipeptide-based multicomponent hydrogels, is evident in various fields, including biomedical devices, photodynamic treatment, and continuous bioprinting. Misra et al. reported on the spontaneous supramolecular assembly of a peptide scaffold that has been modified in its backbone structure. This microstructure of the scaffolds has demonstrated its utility in the immobilization of semiconductor QDs, as depicted in Figure 4. Additionally, it has shown promise in cell culture applications [104]. The peptide scaffold possesses a notable characteristic, whereby it demonstrates a high degree of efficacy in the process of gelation in both aqueous phosphate buffers and aromatic organic solvents. The successful creation of chiral fibrous structures, which enable the transfer of chirality, was achieved through the co-assembly of chiral gelators and chiral FeS_2_ quantum dots (QDs) using noncovalent interactions [105]. The helical pitch and diameter of the co-gels exhibited a high degree of control under the influence of circularly polarized light (CPL). This study not only demonstrates the precise fabrication of chiral composite nano-systems, but also provides insights into the regulation of helical chirality in composite nanomaterials.

## 6. Biomedical Significance Properties of the QDs-Polymer Hydrogels

**Figure 5:**
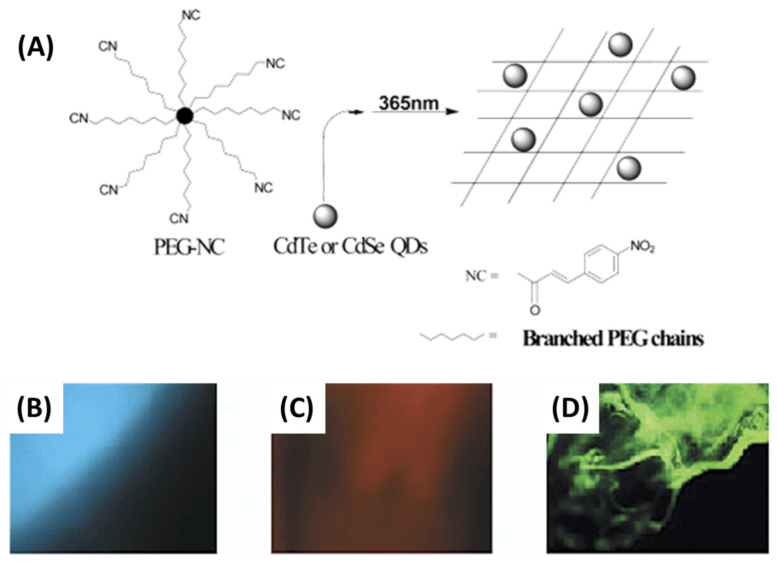
(**A**) Photo-cross-linking from PEG-NC macromer and QD physical immobilization in polymer matrix (epifluorescence pictures of PEG-NC hydrogels (**B**–**D**)). UV-excited. Image: 895 μ 713 μm. Reproduced with permission from [108] © 2003 American Chemical Society.

The incorporation of CDs into the gel matrix leads to distinctive characteristics of the hydrogel, concurrently altering the cross-linked arrangement. The hypothesis was put forth that CDs could potentially improve the effectiveness of hydrogel systems due to their intrinsic fluorescence and low toxicity, operating through a range of mechanisms. Some of the techniques employed in the field involve the immobilization of surface functional groups on CDs to enhance their fluorescence properties, as well as the utilization of CDs as biocompatible carriers, among other strategies. Nevertheless, the integration of CDs as cross-linking agents, either through physical or chemical means, within the gel matrix, leads to a notable improvement in the mechanical characteristics and self-repair capabilities of hydrogels based on CDs. Hydrogels that incorporate CDs exhibit highly captivating luminescent characteristics inherent to CDs [106,107]. There exists empirical evidence supporting the notion that the incorporation of compact discs (CDs) within hydrogel matrices enhances both the intensity and durability of fluorescence, while concurrently mitigating the occurrence of quenching facilitated by agglomeration. The integration of nanocrystalline quantum dots (QDs) into a photo-cross-linked poly(ethylene glycol) (PEG) hydrogel was successfully accomplished, leading to the formation of a hybrid nano-system (Figure 5) that combines both inorganic and organic constituents [108]. 

**Figure 6:**
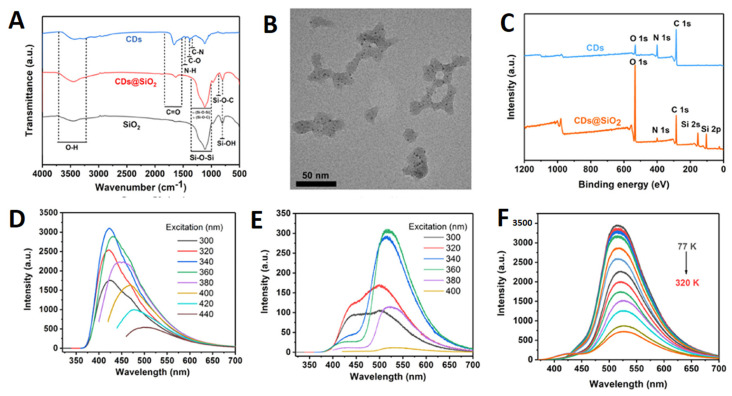
(**A**) FT-IR spectra of CDs, CDs@SiO_2_, and SiO_2_. (**B**) HRTEM of CDs@SiO_2_. (**C**) XPS survey of CDs@SiO_2_. (**D**) Aqueous CDs@SiO_2_ photoluminescence (PL) emission spectra at different excitation wavelengths. (**E**) Aqueous CDs@SiO_2_ excitation wavelength-dependent afterglow emission spectra. (**F**) Solid-state CDs@SiO_2_ afterglow emission spectra at different temperatures under 356 nm excitation. Reproduced with permission from [111] © 2019 American Chemical Society.

The primary advantage of QD-photo-cross-linked immobilization may lie in its potential to facilitate the advancement of more durable photonic materials. One example of a preventive measure is inhibiting the expansion or inducing the structural stability of PEG macromolecules. Matrix modifications can lead to the isolation or aggregation of nanoparticles. In contrast, the cross-linking of a PEG macromolecule can exhibit reversible modifications that are dependent on the wavelength when the nitrocinnamate moiety is employed [109]. Chiral carbonized polymer dots (Ch-CPDs) have garnered significant interest in the scientific community owing to their distinctive photonic characteristics. In their study, Ru et al. documented the achievement of synthesizing red and multicolor-emitting Ch-CPDs through the utilization of a solvothermal technique at a consistent temperature. The precursors employed in this process were L-/D-tryptophan and o-phenylenediamine, which are readily accessible [110]. The phenomenon of room-temperature phosphorescence (RTP) exhibited by CDs has garnered significant attention due to its potential applications in various domains, such as data security, white-light-emitting diode (WLED) technology, and physiological imaging. The task of creating RTP probes with extended emission lifetimes in aqueous environments poses significant challenges. Li et al. presented a practical method for achieving ultralong room-temperature phosphorescence (RTP) in air-saturated aqueous systems by utilizing carbon-dot-based silica composites (CDs@SiO_2_) with emission durations of up to 1.64 s (Figure 6) [111]. CDs@SiO_2_ is an intriguing material for application in the field of biological imaging due to its exceptional phosphorescent properties, compact dimensions, and solubility in water. 

Light emission from carbon dots (CDs) embedded in a silica gel matrix was discovered to occur at room temperature [112]. Malonic acid and ethylene diamine were used as precursors in the synthesis to maximize the abundance of C=O and C=N functionalities on the CDs’ surfaces. CDs emit a brilliant blue fluorescence in an aqueous dispersion, and once absorbed into silica gel, they create a green afterglow that is readily apparent to the naked eye. Phosphorescence tests at 380 nm excitation revealed a lifespan of approximately 1.8 s, the longest yet recorded for CDs in solid-state matrices. Zhou et al. designed a new ratiometric fluorescence sensor based on a CdTe QDs-doped hydrogel optical fiber for sensitive and real-time detection of Fe^3+^ ions [113]. The core-cladding structure and step-index profile of the hydrogel fiber trap light within its core. The hydrogel fiber is loaded with both green-emitting thioglycolic acid-capped quantum dots (gQDs) and red-emitting N-Acetyl-l-cysteine-capped quantum dots (rQDs). The rQDs’ fluorescence is suppressed by the presence of Fe^3+^ ions in the hydrogel matrix, but the gQDs’ fluorescence is unaffected. There has been a lot of focus on developing specialized gelators to enable the production of nanogels with desirable responsive properties. Using the gel properties of nanocellulose (NC) because of its accessibility, sustainability, and renewability, we reported the fabrication of a fluorescent hydrogel based on S,N-co-doped graphene quantum dots (S,N-GQDs) serving as a luminophore and sensitizer [114]. Both GQD solutions and hydrogels containing the GQDs showed enhanced fluorescence. The fluorescent CQDs/hydrogel nanocomposite material (CQDsHG) produced by Wu et al. has good stability and adsorption for detecting Fe^3+^ [115]. The results suggest that combining CQDs and HG for the adsorption and quantitative detection of heavy-metal ions in an aqueous environment can improve the performance of both components. It has been reported that an agarose hydrogel anchored with carbon dots (CD) can be used as a thin-film solid sensing substrate for the optical detection and efficient ion separation of quintet heavy-metal ions [116]. The presence of a prominent peak at 206 nm (typical of chitosan) in the UV-visible spectra of Agr/CD, but not Agr, provided further evidence for the presence of a chitosan moiety in the Agr/CD hydrogel network. A photoluminescence (PL) analysis was then performed on the hydrogel films using excitation wavelengths of 340, 360, 380, 400, 420, and 440 nm. At an excitation wavelength of 360 nm, the PL intensity was greatest in both hydrogel films. According to Kharlampieva et al.’s description, responsive photoluminescent hybrid materials with immobilized QDs were fabricated using spin-assisted layer-by-layer assembly [117]. Thioglycolic acid-stabilized CdTe nanoparticles can be contained in a poly(allylamine hydrochloride) (PAH) or poly(sodium 4-styrenesulfonate) (PSS) polyelectrolyte, while a poly(methacrylic acid) (PMAA) hydrogel matrix provides an elastomeric network with pH-responsive properties. Quantum dot layers are sandwiched between PSS-PAH bilayers in this hybrid hydrogel matrix. The system’s photoluminescent intensity changes in a pH-dependent and reversible fashion. The photoluminescent intensity of the hybrid matrix is reduced when there is an excess of negative charge at a high pH, while it is greatly enhanced when there is an excess of positive charge at a low pH. Hydrogel-LbL assemblies containing QDs open the door to a novel approach to the construction of materials with pH-triggered optical characteristics that might be further developed into pH- or chemical-sensing devices. Furthermore, the author demonstrated a novel 3D porous fluorescent hydrogel composed of CDs and cellulose nanofiber (CNF) [118]. The n-π* transition of the carbonyl group from the CDs may be responsible for the single, distinct peak at 355 nm in the UV-vis absorption spectra of the porous hydrogel. Both excitation at 350 nm and emission at 450 nm were found to be optimal. In addition, a new, low-cost fluorometric platform was established by embedding sulfur- and nitrogen-co-doped graphene quantum dots in nanocellulosic hydrogels and observing the PL emission spectra of the resulting luminescence. Hydrogels that emit light were made with a straightforward aqueous method (acrylic acid) by incorporating lanthanide ions and carbon dots (CD) into a network of polyacrylamide and poly(vinyl alcohol). The ratio of CD (which produces blue light) to lanthanide ions (which produce green and red light) was optimized to create white luminescence [119]. The luminous hydrogel displayed a chromic response to several stimuli, such as pH, organic vapors, transition-metal ions, and temperature, by capitalizing on the combined specific sensitivities of the many emitters. The hydrogel’s white-light emission and stretchability were further shown by a 400% fracture strain. Hydrothermal synthesis of ammonium hydrogen citrate was used to produce the carbon dots (CDs). The solvent-casting technique was used to create the chitosan-based nanocomposites with varying concentrations of CDs [120]. The chitosan/CDs nanocomposite prepared with a concentration of 1.0 wt% carbon dots held the best optical, biological, and mechanical features. The results further show that the suggested chitosan/CDs nanocomposites exhibited remarkable pH-sensitive characteristics. For the detection of miR-21 in MCF-7 cancer cells, Mohammadi et al. created carbon dots-chitosan nanocomposite hydrogels by reacting carbon dots generated from different aldehyde precursors with chitosan that had been functionalized with the ssDNA probe [121]. Moreover, the Schiff-base reaction (creating imine bonds) between the amine in chitosan and aldehyde groups on the surface of the CDs was used to create three luminous hydrogels. Such hydrogels are easily generated in aqueous solution by creating an imine link between chitosan and aldehyde functional groups of the CDs’ surface. Hydrogen bonding allows for the formation of chitosan-CDs-EDTA complexes, which are comprised of chitosan molecules with -NH_2_/-OH groups, CD molecules with -COH groups, and ethylenediaminetetraacetic acid (EDTA) molecules with -COOH groups.

## 7. Biosensing Applications

Hydrogels are crucial in a variety of scientific and medicinal applications. Their application in bioanalysis and sensing is growing in importance. In this section, we look at hydrogels in general and talk about the different ways they can be used. Hydrogels have many applications beyond biomolecule immobilization and embedding, such as a responsive material, a component of wearable devices, and a functional material. Biosensing is a state-of-the-art medical technique that makes use of biosensors, which are devices with varying degrees of sensitivity to various biological substances. Nanotechnology in biosensing has piqued the interest of scientists and medical professionals due to its many advantages, including high solubility, minimal toxicity, and good biodegradability. QDs-hydrogel composites are an advanced immobilization material that can improve the biosensor performance in challenging environments.

**Figure 7:**
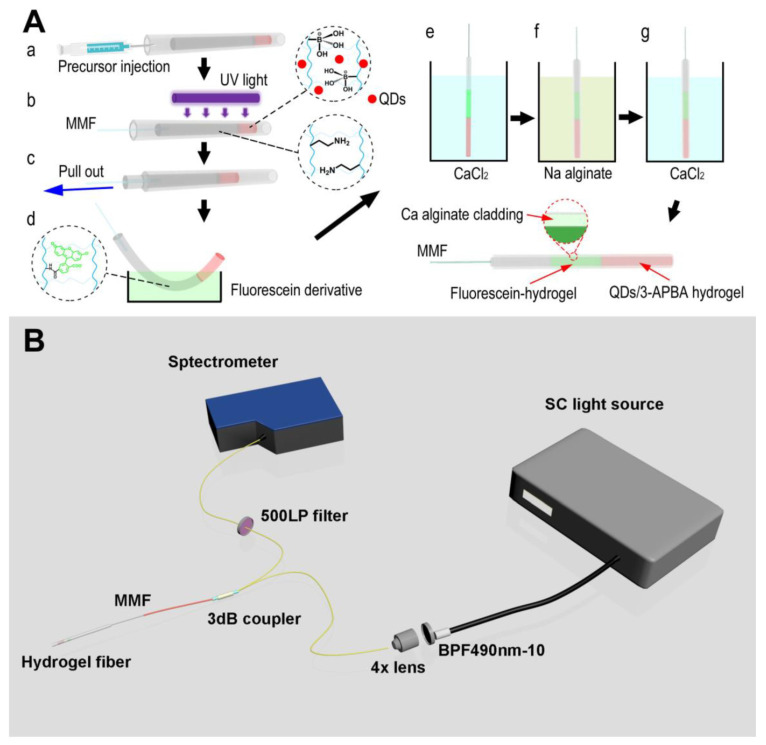
(**A**) Fabrication of difunctional fluorescent hydrogel fiber. (**B**) Optical setup for simultaneous continuous pH and glucose monitoring. Reproduced with permission from [129] © 2022 MDPI.

Hydrogels can be made fluorescent by using a variety of luminescent materials, such as organic fluorescent dyes (OFDs) or rare-earth elements (REs) [122]. However, the inclusion of hydrophobic OFDs typically results in hydrogels with compromised mechanical properties [123]. Despite hydrogels based on REs having enhanced fluorescent features, higher transparency, and mechanical qualities, the possible biotoxicity of REs may limit their biological usage [124]. Hydrogels comprising alginate and cellulose nanofibers (CNFs) were surface-functionalized with biomass CQDs produced from glucose, xylose, and glucosamine, and a variety of all-biomass fluorescent hydrogels was exhibited. Strong fluorescence properties and considerably improved mechanical capabilities were found in composite hydrogels cross-linked with CQDs [125]. To identify Fe^3+^ and Au nanoparticles (NPs) in water, researchers used a biomass-fluorescing hydrogel for the first time as a solid sensing platform. Results from this study point to biomass hydrogels as a potentially useful material for applications in biomedical imaging, biosensing, and monitoring. One can use a ratiometric fluorescent approach based on CD and Rh6G fluorophores for glucose detection, employing a bi-enzyme (GOx and HRP) system in either an aqueous solution or a solid-state PAA film cross-linked with DAPEG [126]. Microarrays (QDs) were created by photopatterning a solution containing poly(ethylene glycol) (PEG) hydrogel, poly(ethylene glycol) diacrylate (PEG-DA), photo-initiator, tyrosinase, and CdSe/ZnS QDs. Tyrosinase and QDs were trapped in hydrogel microarrays after photo-induced cross-linking, making them fluorescent and phenol-responsive. The tyrosinase that was trapped in the hydrogel microarray oxidized phenol to quinones, which dampened the fluorescence of the QDs. The hydrogel microarray fluorescence intensity linearly decreased with the increasing phenol content, and a detection limit of 1.0 M was established for this system. Several aldehyde precursors have been used to create carbon dots-chitosan nanocomposite hydrogels [121]. A ssDNA probe was added to the test to find microRNA-21 in MCF-7 cancer cells. Three luminous hydrogels were made by mixing the aldehyde groups on the surface of the CDs with the amine in the chitosan. Hydrogel sheets, CDs, and CDs-chitosan nanocomposite hydrogels were also described by their UV-vis absorption and fluorescence spectra. Yuan et al. made a CdTe QD gel that holds enzymes and can be used as a flexible base for biosensors [127]. Sol–gel switching was also seen in the QD gels as they were made, and the gelation time of the CdTe QDs was reduced to a few days by dissolving the mercaptosuccinic acid (MSA)-capped CdTe QDs in a neutral phosphate buffer (PB). Additionally, CdTe QD hydrogels were made that contained the enzyme tyrosinase (TRS) and were used for biosensing dopamine. Enzyme bioreceptors (such as glucose oxidase or horseradish peroxidase) and fluorescent reporters have been placed inside a self-assembled peptide hydrogel made of Fmoc-diphenylalanine and used as a biosensing platform (e.g., CdTe and CdSe QDs) [128]. Mixing a solution of QDs and enzymes in water with a solution of a monomeric peptide (Fmoc-diphenylalanine) was all it took to physically fix them in place in the hydrogel matrix in a single step. They were able to find analytes such as glucose and dangerous phenolic chemicals in the peptide hydrogel by using the hybridized QDs to stop the photoluminescence. The Michaelis–Menten constant (KM) of the photoluminescent peptide hydrogel was found to be 3.12 mM (GOx for glucose) and 0.82 mM (HRP for hydroquinone) when compared to other gel materials. Based on these results, peptide hydrogels could be used as an alternative optical biosensing platform because they are good at encapsulating fluorescent reporters and bioreceptors, are easy to make because they self-assemble, and allow target analytes to easily move through them. In order to properly diagnose and treat diabetes, it is crucial for patients to be aware of their current blood glucose levels in real time. Therefore, studying continuous glucose monitoring (CGM) is essential since it provides us with real-time data on the dynamic changes in our health (Figure 7). It has been reported that pH and glucose can be constantly monitored using a novel hydrogel optical fiber fluorescence sensor that is segmentally functionalized with fluorescein derivative and CdTe QDs/3-APBA [129]. Complexation of PBA and glucose in the glucose-detecting section will cause local hydrogel expansion and a decrease in quantum dot fluorescence. Hydrogel optical fibers allow for real-time transmission of fluorescence to a detector. The dynamic change in the glucose concentration can be tracked since both the complexation reaction and the swelling–deswelling of the hydrogel are reversible. A change in the protolytic form of fluorescein connected to another part of the hydrogel can be used to detect a shift in pH. Since the reaction between PBA and glucose is pH-dependent, measuring pH is important as a means of compensating for pH mistakes in glucose detection. There is no signal interference between the two detecting units because their emission maxima are at different wavelengths (517 nm and 594 nm, respectively). The sensor has a pH range of 5.4 to 7.8 and a glucose range of 0 to 20 mM. This sensor’s benefits include the ability to detect multiple parameters at once, to integrate transmission and detection, to detect dynamically in real time, and to be biocompatible. Several study groups are working hard to make fluorescent hydrogel biosensors that can detect things in different ways (Table 2).

The hydrogel microstructure arrays used in the microfluidic devices developed by Jang et al. serve to entrap QD-enzyme conjugates [130]. Based on the idea that H_2_O_2_, produced by various oxidase-catalyzed processes, may easily quench the photoluminescence of QDs, research was conducted using QDs conjugated with glucose oxidase or alcohol oxidase for the detection of glucose and alcohol, respectively, in microfluidic devices. In order to make a fluorescent hydrogel microarray that was sensitive to glucose or alcohol, the model oxidase enzymes, glucose oxidase (GOX) and alcohol oxidase (AOX), were entrapped inside the hydrogel microstructures and connected to carboxyl-terminated CdSe/ZnS QDs. Encapsulated GOX and AOX induced glucose and alcohol oxidation, respectively, to produce H_2_O_2_, which in turn dimmed the fluorescence of the connected QDs. This method has a detection limit of 50 M for glucose and 70 M for alcohol because the fluorescence intensity of the hydrogel microstructures decreases with the increasing concentrations of glucose and alcohol, respectively. Since each microchannel in our novel microfluidic system could conduct independent experiments, we were able to detect glucose and alcohol at the same time. Won et al. developed hydrogel biosensors for cancer detection using ureidopyriminone-conjugated gelatin (Gel-UPy) and di-selenide-containing carbon dots (dsCD). When di-selenide groups of the dsCD inside hydrogels are cleaved by glutathione (GSH) or reactive oxygen species (ROS), the hydrogels’ self-healing, conductivity, and adhesiveness are all altered [131]. Gel-UPy/dsCD hydrogels demonstrate significantly quicker healing in tumor circumstances (MDA-MB-231), in comparison to physiological situations (MDCK). The breakdown of di-selenide bonds is another way in which dsCD degradation affects electrochemical signals. High concentrations of GSH or ROS in hydrogels give them excellent adhesive qualities, allowing them to detect cancer in living beings.

**Table 2:** Different types of chemo-sensors and their detection limits and targeting analytes.

Sensing Target	Sensor	Gel Matrix	Detection Limit	Ref.
Cr(VI)	CDs	Lignin+CNF	~11 mg/L	[132]
Cr(VI)	CDs	PVP	1.2 µM	[133]
Ag^+^	CDs	BMIM-BF_4_ IL	0.55 µg/mL	[98]
Fe^3+^	CDs	MCC	65 µM	[134]
NO_2_^-^	Doped CDs	Agarose	0.018 µM	[135]
TC	CDs	Sodium alginate	2 µM	[136]
Glucose	CDs	PAA	0.04 µM	[125]
*Bacillus* and *Staphylococcus* strains	CDs	DTG	10^5^ cells/mL	[137]
Progesterone	CdSe/CdS/ZnS	PEG	55 µM	[138]
Dopamine	CdTe	MSA	50 nmol/L	[127]
Uric acid	CdS	GPTMS	50 µM	[139]
Fe^3+^	CdTe	PEGDA+ HMP	14 nM	[113]
Polyaromatic compounds	GQDs	AETA+MBA	-	[140]
Laccase	S,N-codoped GQDs	NC	0.048 U/mL	[141]

CDs, carbon dots; CNF, cellulose nanofiber; BMIM-BF4, 1-butyl-3-methylimidazolium tetrafluoroborate; IL, ionic liquid; PVP, polyvinylpyrrolidone; TC, tetracycline; DTG, 6-O-(O-O′-dilauroyltartaryl)-D-glucose; PEG, poly(ethylene glycol); MSA, mercaptosuccinic acid; GPTMS, 3-glycidoxypropyl trimethoxysilane; PEGDA, polyethyleneglycol diacrylate; HMP, 2-hydroxy-2-methyl-propiophenone; GQDs, graphene quantum dots; MBA, N,N′-methylenebisacrylamide; NC, nanocellulose.

**Figure 8:**
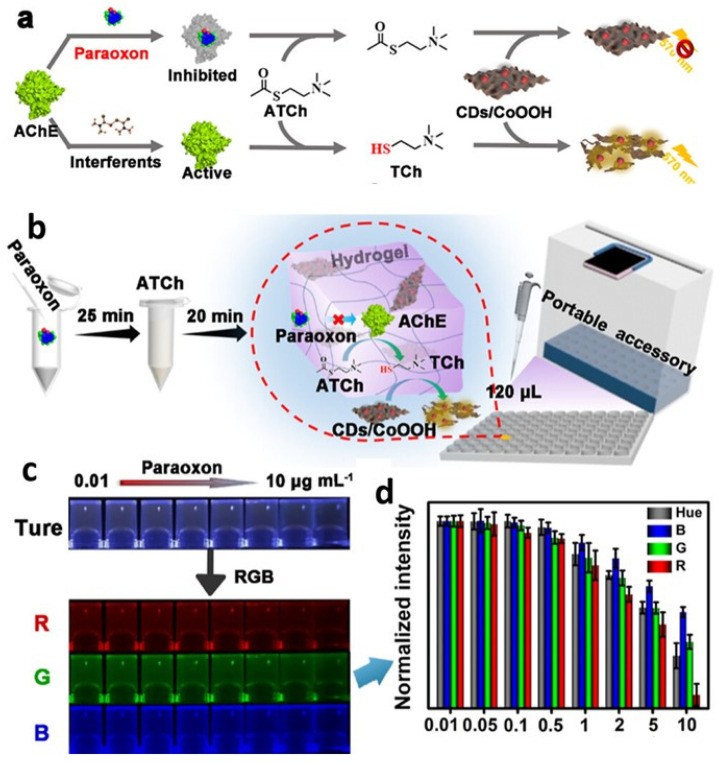
(**a**) Illustration of a strategy for paraoxon detection. (**b**) The paraoxon hydrogel kit is schematically depicted. (**c**) The kit’s true color graphics are separated into red, green, and blue components. (**d**) The ImageJ program is used to digitize the normalized intensity. Reproduced with permission from [150] © 2022 American Chemical Society.

It is critical to develop a rapid and accurate method for detecting lactate since a high lactate concentration is a useful indicator in clinical diagnosis. Hydrogel microspheres containing QDs were developed for the speedy, on-the-go detection of lactate in bodily fluids [142]. Hydrogel microspheres (Alg@QDs-LOx MSs) were created by enclosing CdTe QDs and lactate oxidase (LOx) in alginate hydrogels (Alg). Increased storage stability for lactate oxidase and anti-interference characteristics are demonstrated by the Alg@QDs-LOx MSs, which exhibit a dense multi-layer network topology. In as short as 15 min, fluorescent microspheres can distinguish between lactate concentrations as high as 2.5 mM in human serum. It is more convenient and less expensive than traditional methods because the results can be seen with the naked eye or tracked on smartphones, rather than requiring expensive, specialized equipment. The cutoff for detecting lactate is 1.25 M. Bui et al. developed an optical cholesterol biosensor using carbon dots and hemoglobin (CD/Hb) [143]. This optical sensor detects cholesterol by amplifying CD’s fluorescence, which is normally inhibited by interactions between CD and Hb in the CD/Hb complex. CD is dissociated from the CD/Hb complex because the hydrophobic contacts between Hb and cholesterol are more advantageous than the interactions between CD and Hb. The CD/Hb combination enabled selective detection of cholesterol across a linear range of 0 to 800 M in human blood plasma, with a limit of detection of 56 M and a reaction time of 5 min. Cholesterol detection using a CD/Hb complex-based biosensor is simple, highly sensitive, selective, rapid, non-destructive, and cost-effective. As a simple, low-cost agent for visual assessment of intracellular and serum glucose levels utilizing a multimodal photoluminescence (PL) bio-platform, R-CDs/B_2_O_3_ were developed by dispersing rhodamine B carbon dots (R-CDs) in a matrix of boric acid (BA) [144]. Due to the strong linear correlation between the R-CDs/B_2_O_3_ PL spectra and glucose levels, the latter can be monitored in real time in vitro. By observing the gel’s fluorescent color changes under a UV light and processing the green channel intensities of the colorful photos with the smartphone’s RGB option, it was also demonstrated that R-CDs/B_2_O_3_ may be used as a portable test gel for blood glucose. The ability for persons with diabetes to monitor their own blood sugar levels is a huge benefit. Serum glucose levels were also accurately assessed with this method. A highly sensitive and selective thrombin biosensor based on a carbon nanocomposite with an aptamer-recognition surface was recently published [145]. This biosensor (PQdot) was developed by modifying screen-printed carbon electrodes (SPCE) with a nanocomposite of fullerene (C60), multi-walled carbon nanotubes (MWCNTs), polyethylenimine (PEI), and polymer QDs. The synergistic effects of the C60/MWCNTs-PEI/PQdot nanocomposite are the result of the complementary properties of the individual nanoparticle components. Polymer QDs also exhibit beneficial properties, including high stability, high surface-to-volume ratio, high electrical conductivity, high biocompatibility, and high mechanical and chemical stability. The high amine content of the C60/MWCNTs-PEI/PQdot nanocomposite made it an appropriate substrate for the covalent immobilization of amino-linked aptamer (APT), which improved the sensitivity and stability of the aptasensor. Differential pulse voltammetry (DPV) was used to conduct the measurements with a probe solution. The binding of thrombin protein to aptamers immobilized on the transducer reduces electron transport at the electrode/electrolyte interface, hence lowering the peak current (IP) in DPV. A superabsorbent hydrogel biosensor for blood glucose was created using nanoparticles of gum tragacanth (GT), acrylic acid (AA) monomer, N,N′-methylenebisacrylamide (MBA), and fluorescein O,O′-diacrylate (FlA-DA) [146]. The enzyme glucose oxidase (GOx) was used to create a bioreceptor that could be immobilized in hydrogels. The biosensor’s robust fluorescence could be attributed to the QDs and FlA-DA’s synergistic action. The biosensor oxidized glucose using an enzyme, producing hydrogen peroxide that blocked fluorescence. The rate of glucose quenching was associated with glucose concentrations. The proposed system’s 0.1 mM detection limit and 1.35 mM Michaelis–Menten constant (KM) are both much lower than those of conventional gel materials. Furthermore, this cutting-edge technology has shown impressive reproducibility and accuracy with raw, clinical blood samples. Based on the results, it appears that this newly created, highly fluorescent biosensor is a promising diagnostic tool due to its simple production process and efficient immobilization of fluorescent transmitters and bioreceptors. Using gum tragacanth (GT) polysaccharide nanoparticles (50 nm) with immobilized CdTe QDs and glucose oxidase (GOx), a highly functionalized superabsorbent nanohydrogel was created [147]. The developed biosensor was able to enzymatically detect blood glucose levels in real serum samples with high reproducibility and accuracy. This superabsorbent nanohydrogel matrix may find application as a diagnostic tool due to the efficient immobilization of QDs and GOx. Carbon dots (CDs) and their doped counterparts, such as nitrogen-doped CDs (N@CDs), have been synthesized from a wide range of precursors using both bottom-up and top-down approaches. Nanoscale 2D carbon-based materials are on the rise due to their advantageous combination of biocompatibility, manufacturing simplicity, water dispersibility, and prospective applications. It was also considered whether or not a biocompatible modification may be used to induce efficient defect sites in GQDs [148]. The effectiveness of graphene quantum dots (GQDs) in biosensing and microbiological applications was investigated by applying lithium salt of 6-aminohexanoic acid (caproic acid), a well-known modifier for graphene and carbon nanotube dispersions. By changing the nanomaterials’ pH and charge through adsorption, these modifiers can potentially enhance their sensing abilities for GQDs and antibiofilm qualities. The effectiveness of the modified GQDs at eliminating *Staphylococcus aureus* biofilms was evaluated using dopamine sensing. If PEC active material and biological receptors are combined, photoelectrochemical (PEC) performances may suffer from a shortage of accessible surface areas. In order to solve this problem, Hao et al. introduced 3D nitrogen-doped graphene hydrogel (3DNGH) into PEC sensing for the first time [149]. Nitrogen incorporation into the graphene framework not only improved the conductivity beyond that of 3D graphene, but also allowed for a high loading amount of PEC active nanomaterial and biological receptors. ZnO/3DNGH was created during the hydrothermal process. ZnO/2DNG considerably outperformed ZnO/3DGH in terms of the photocurrent when exposed to visible light due to its enhanced light absorption and faster charge transfer. ZnO/3DNGH was used to immobilize the enzymes, and the resulting PEC biosensing platform demonstrated potential. According to the results of this study, 3DNGH is a great place to conduct future investigations involving PEC biosensors in the fields of medicine and environmental science. To guarantee food safety, it is essential to create portable, measurable, and user-friendly sensors for on-site monitoring of organophosphorus pesticides (OPs) (Figure 8) [150]. Fluorescent hydrogel sensors based on a carbon dot/cobalt oxyhydroxide (CD/CoOOH) composite are fabricated as a handcrafted, portable supplemental device for precise OPs measurement. As a fluorescent signal indication, an agarose hydrogel kit encloses an orange-emitting CD/CoOOH composite, where the signal can be enhanced for detection, shielded from interference, and made more stable. The substrate is hydrolyzed by acetylcholinesterase (AChE), yielding thiocholine, which initiates the breakdown of CoOOH, hence enhancing the hydrogel platform’s fluorescence. OPs can specifically decrease AChE activity, which in turn reduces thiocholine production and platform fluorescence.

## 8. Drug Delivery Applications

Hydrogels with embedded QDs are an exciting new technology for improving drug delivery. Hydrogel matrices can be used to create targeted and controlled release systems by enclosing QDs containing medicines inside. Hydrogels provide a biocompatible and stable environment, while QDs’ distinctive optical and electrical properties allow for real-time monitoring of drug release processes. Targeted distribution to sick cells or tissues is enabled by functionalizing the hydrogel surface with targeted ligands [151]. The use of QDs in tandem with hydrogels shows significant promise for improving drug delivery efficiency and specificity, opening up new avenues for treatment.

Hydrogels are able to influence the microenvironment of a tissue thanks to their porous and hydrated molecular structure [152]. Chemical or physical cross-linking is used to create hydrogel networks [19]. Under physiological conditions, a three-dimensional hydrogel network made from highly hydrated polymers may absorb environmental water and swell several times without dissolving [153]. Using doxorubicin (DOX) as a pharmacological model with broad-spectrum anticancer capabilities, graphene quantum dot (GQD) as a nanoparticle was incorporated into carboxymethyl cellulose (CMC) hydrogel to create unique hydrogel nanocomposite films with anticancer qualities [154]. DOX-loaded CMC/GQD nanocomposite hydrogel films were tested against K562 blood cancer cells by the MTT assay and drug release investigations at two different pH levels. The produced CMC/GQD nanocomposite hydrogel films were non-toxic to blood cancer cells (K562) and exhibited enhanced in vitro swelling, breakdown, water vapor permeability, and pH-sensitive drug transport capabilities. Based on the data, these nanocomposite hydrogel films can be offered for usage as a drug delivery system and anticancer film. Singh et al. developed a DNA-CDs hybrid hydrogel by taking advantage of the low extracellular pH found in the tumor microenvironment. Hydrogels were used as a container for the sustained release of the anticancer medication doxorubicin (DOX) and CDs [155]. Changing the pH of the solution from alkaline to neutral induced a visible sol–gel transition in the CD-DNA hybrid hydrogel. Drug release from hydrogel was examined in vitro for its dependence on both time and pH. Hydrogel was stable for a month at physiological pH, but completely dissolved and released drug molecules over 10 to 11 days in acidic pH, mimicking the tumor microenvironment. HeLa cells were subjected to a cell viability assay, and the results showed that the cells were killed slowly but effectively in the presence of DOX-laden hydrogel due to the acidic pH being optimal for hydrogel disruption. In order to produce hydrogels that are both biodegradable and biocompatible, GQDs were proposed as a unique and safe crosslinker for carboxymethyl cellulose [156]. To quickly and easily prepare the CMC/GQDs films, casting was used. The CMC/GQDs increased the tensile strength, accompanied by pH-sensitive swelling and breakdown. The produced CMC/GQDs nanocomposite was further analyzed for its fluorescent characteristics to determine whether or not it may be used in fluorescent bioimaging. We used the anticancer medication doxorubicin (DOX) to investigate the drug delivery properties of CMC-GQDs. In vitro cytotoxicity tests were performed on HT29 human colon cancer cells. The produced nanocomposite hydrogel showed promise as a pH-triggered, site-specific drug delivery system due to its biocompatibility and pH-sensitive drug delivery behavior with the CMC/GQDs.

## 9. Emerging Trends and Future Directions for Research

The application of hydrogels that incorporate quantum dots has emerged as a highly promising strategy in the realm of sensing applications. The primary cause of this phenomenon can be attributed to the unique optical properties displayed by quantum dots, along with the versatile nature of hydrogel matrices. One of the notable trends observed in this specific field relates to the progress of highly sensitive sensors, achieved through the integration of quantum dots into hydrogel networks. The fluorescence properties of quantum dots are contingent upon their size, allowing for the manipulation of the emitted wavelength and the attainment of greater brightness when compared to traditional organic fluorophores. This specific characteristic enables the advancement of sensors that demonstrate improved sensitivity and detection thresholds. The incorporation of quantum dots into hydrogels has been found to be effective in detecting various analytes, as demonstrated in recent scientific studies. An instance of a noteworthy progression in the domain pertains to the creation of pH-responsive hydrogels that are combined with quantum dots. This integration enables the ongoing and instantaneous monitoring of pH fluctuations within biological systems. The potential applications of these sensors encompass a wide range of fields, including disease diagnosis, environmental monitoring, and food safety. The integration of quantum dots with temperature-sensitive hydrogels has demonstrated promising capabilities for temperature-sensing applications. This technology exhibits potential for diverse applications, encompassing biomedical engineering and energy management. 

A growing trend in the field of hydrogels based on quantum dots for sensing purposes pertains to the progress made in developing ion-selective sensors. The integration of ion-responsive receptors into the hydrogel matrix enables researchers to develop sensors with a high degree of selectivity for ion detection. Quantum dots exhibit remarkable efficacy as reporters in these sensors, providing a highly sensitive and reliable method for detecting changes in the ion concentration. The sensors exhibit considerable potential for application in the domains of environmental monitoring, industrial processes, and biomedical diagnostics.

Furthermore, there has been a growing interest in the utilization of hydrogels containing quantum dots in conjunction with biomolecules for the purpose of sensing. Through the process of functionalizing the surface of hydrogels with biomolecular receptors, such as antibodies or enzymes, scientists have the capability to create biosensors that exhibit a high level of specificity in detecting biomarkers or pathogens. The integration of quantum dots significantly improves the sensitivity of these biosensors, thereby facilitating the detection of analyte concentrations at low levels. The utilization of this technology has considerable ramifications within the domains of healthcare, disease diagnostics, and personalized medicine. In addition to their utility in sensing applications, hydrogels incorporating quantum dots exhibit significant promise in the realm of pharmaceutical administration. Extensive research has been conducted on hydrogels as potential drug carriers owing to their remarkable biocompatibility, substantial water content, and adjustable drug release characteristics. The objective of researchers is to enhance the functionality of smart drug delivery systems by integrating quantum dots into hydrogel matrices.

An avenue for further investigation involves the advancement of stimuli-responsive drug delivery systems utilizing hydrogels based on quantum dots. These systems have the capability to administer drugs upon encountering specific stimuli, such as alterations in pH, temperature, or the existence of enzymes. Quantum dots offer a viable approach for real-time monitoring of drug release, enabling accurate regulation of drug dosage and delivery kinetics. These systems possess the capacity to significantly transform the field of drug delivery, facilitating the implementation of individualized and precise therapeutic approaches. In addition, the incorporation of quantum dots into hydrogels presents opportunities for the integration of therapeutic and imaging modalities. Quantum dots exhibit exceptional optical characteristics, rendering them highly suitable for various imaging applications. Through the integration of imaging agents into hydrogel carriers, researchers have the ability to create theranostic platforms that enable the simultaneous delivery of drugs and imaging capabilities. The integration of this system facilitates the continuous monitoring of drug distribution and effectiveness, resulting in enhanced treatment outcomes and minimized adverse effects.

In summary, hydrogels incorporating quantum dots have emerged as a highly promising platform for applications in sensing and drug delivery. The distinctive optical characteristics exhibited by quantum dots, in conjunction with the adjustability and biocompatibility of hydrogels, present significant prospects for the advancement of exceptionally responsive sensors and intelligent pharmaceutical delivery systems. Future research in this particular domain ought to prioritize the continued enhancement of quantum dots-based hydrogels’ performance, as well as the exploration of innovative applications. Additionally, efforts should be made to effectively implement these technologies in practical contexts, such as healthcare, environmental monitoring, and other pertinent fields.

## 10. Conclusions

There is a lot of room for growth in the biological applications of nanocomposite polymeric gels incorporating fluorescent nanoparticles. Combining polymeric gels, which are both adaptable and biocompatible, with fluorescent nanoparticles, such as quantum dots, opens up exciting new avenues for biological imaging, sensing, and drug delivery. In addition to the enhanced sensitivity and tunability in the emission wavelength, these nanocomposite gels enable real-time monitoring, targeted therapy, and imaging-guided interventions. Fluorescent nanoparticles impregnated into nanocomposite polymeric gels provide for highly sensitive and specific imaging capabilities, allowing for precise visualization and characterization of biological structures and processes. Fluorescent nanoparticles’ tunable emission wavelengths allow for multiplexing, which improves diagnostic capabilities by allowing simultaneous imaging of numerous targets. Being able to view and track individual molecules or cells within a biological context has significant implications for disease diagnosis, treatment monitoring, and a greater understanding of biological mechanisms at the cellular and molecular levels. The sensing capabilities of these systems can be further enhanced by including fluorescent nanoparticles into polymeric gels. Integrating customized receptors or probes onto the surface of the nanocomposite gels allows researchers to develop highly sensitive and selective sensors for detecting a broad spectrum of analytes. Fluorescent nanoparticles, with their unique optical properties, combined with the adaptability of polymeric gels have made real-time monitoring and analysis of analytes in complex biological matrices conceivable. This discovery opens up vast opportunities for usage in domains such as environmental monitoring, point-of-care diagnostics, and biomedical research. Nanocomposite polymeric gels with luminous nanoparticles for tailored drug delivery also show therapeutic promise. Drug dosage, release kinetics, and localization can all be precisely controlled by encapsulating therapeutic substances within these gels and triggering their release in response to external stimuli. The integration of fluorescent nanoparticles not only enables simultaneous imaging and therapy but also permits real-time monitoring of medication release and distribution. This theranostic strategy has significant promise for personalized medicine because it can lead to more effective treatment plans with fewer adverse effects.

Nanocomposite polymeric gels impregnated with fluorescent nanoparticles have a bright future in biological applications, but they also come with a variety of challenges and factors to take into account. These include minimizing the gels’ potential toxicity while keeping their biocompatibility and long-term stability intact. These technologies need to go through rigorous preclinical and clinical testing to ensure their safety and efficacy before they can be implemented in actual biological settings. Incorporating fluorescent nanoparticles into nanocomposite polymeric gels, as we have seen, is a unique approach, with huge potential for application in the biomedical industry. By fusing the malleability of polymeric gels with the distinctive properties of fluorescent nanoparticles, scientists have developed state-of-the-art systems for imaging, sensing, and targeted drug delivery. Research and development in this area are ongoing, with the goal of overcoming challenges and improving the functionality of such systems in order to improve patient care and outcomes.

## Figures and Tables

**Table 1:** Different types of fluorescent-nanoparticle-loaded hydrogel synthesis methods.

Fabrication Method	Classification	Advantages	Disadvantages	Refs.
Sol–gel method	Chemical and physical	High purity, precise control over nanoparticle size and distribution, low cost	Long synthesis time, difficult to scale up	[71]
Emulsion polymerization	Surfactant-mediated and Surfactant-free	High yield, fast synthesis time, versatile in terms of polymerization conditions	Poor control over particle size, low stability, high surfactant content	[72,73]
Electrospinning	Coaxial and single jet	Precise control over fiber morphology, good mechanical properties, versatile in terms of polymer selection	Limited to fiber formation, limited control over nanoparticle distribution	[74]
In situ polymerization	Radical-triggered and ionic polymerization	Easy to scale up, good control over particle size and distribution	Limited control over particle morphology, may require toxic solvents	[75,76]
Layer-by-layer assembly	Polyelectrolyte-based and hybrid	Versatile, precise control over nanoparticle distribution and thickness, easy to incorporate functional groups	Time-consuming, requires careful optimization of conditions	[77,78,79]
